# Physiology

**Published:** 1875-08

**Authors:** W. H. Jackson


### PHYSIOLOGY.

BY W. H. JACKSON.

*Read before the Mich. State Dental Association.*

Physiology treats of the laws of life, and the functions of living beings.

Dalton Says, "These peculiar phenomena, by which we so readily distinguish living organisms from inanimate substances, are called vital phenomena, or the phenomena of life."

In order to understand the laws of life, there must be a thorough investigation of all its phenomena; for through the knowledge of the phenomena only are we to discover and come to an understanding of the law; hence we can not make or set up our own ideas as laws regardless of phenomena; if we do we only erect them to be torn away as soon as erected.

To observe phenomena requires an accurate knowledge of anatomy, histology and chemistry.

The human system is composed of numerous organs; each of which performs its own peculiar action or office. This is function.

Scientific men, until recently, stopped here; considering that it was sufficient to understand the number of different organs, their action and the product of their action.

A few restless spirits, which we call workers, not feeling satisfied, have gone deeper, opening up a world of study in the more minute structures of the different organs and tissues of the body. This is histology. Histology and physiology go hand in hand, as one foot aids the other in locomotion.

The microscope has been the stimulator toward this minute investigation, and the revelation which it makes in what we might almost call the atomic world, is truly wonderful; without its revealing powers I should be unable to bring the few remarks I have to make before you; and through its revelation we find that the living human organism, which we see moving about, is not an unit of life, but a multitude of infinitely small units; some just springing into life, some in youth, others in the full vigor and strength of middle age, while still others are ready to die and be cast aside, like the beautiful autumn leaves that lie scattered on every side, giving evidence of their former glory, yet soon to be obliterated, leaving no trace of their previous existence.

Each class of units has its own function to perform, and differs from that of every other in accordance with the tissue it has to build up. One class builds muscle, another cartilage and so on through all the different tissues of the body.

That which is now known as an unit of life, is termed a cell, by this we understand that there is "living or germinal matter, with formed material upon its surface." It is to this cell life I wish to call your attention. "The living or germinal matter is a jelly-like substance, possessing within itself the power of motion, while formed material has not that power and is passive. To be sure formed material, is governed by certain laws and acts in accordance with the action of certain forces which may be brought to bear upon it. This formed material is tissue.

"There are three things necessary to the production of tissue. First, living matter; second, formed material; third, pabulum. All tissue has been of the non-living pabulum, then is taken up and is a part of the living or germinal matter and thrown out as formed material. This formed material having none of the characteristics of the pabulum or the germinal matter which formed it, "it has a character and form peculiar to itself, which has been stamped upon it by the parent builder, the living or germinal matter.

The function of the cell is to build tissue and reproduce cells that shall fill its place when it is no longer able to perform its functions.

The cells that build dentine are called odontoblasts; they first form a thin layer of dentine on the inner surface of the connective tissue between the organ which builds dentine and the organ which builds enamel. The dentification commences at the apex of the cusps; for the incisors and cuspids, one center each; bicuspids, one to two centers each; molars, three to four centers each.

The odontoblasts as fast as they form dentine, recede toward the pulp, that is toward where the pulp cavity is to be formed, small filaments reaching back to the original starting point, forming the dental tubuli.

The enamel cells cover the outer surface of the connective tissue, and begin to deposit the enamel soon after the layer of dentine has commeneed to be formed on the inner surface of the tissue, they commence to build at the highest point of the cusps radiating from that point. Where there is more than one center they spread until they come in contact, the points of contact form what are known as fissures in the crowns of the teeth. All of this hard enamel substance is taken up from the pabulum by the living matter in these little enamel cells; so these little masses of germinal matter build the hard hexagonal rods that vie with the crystal for beauty, sometimes wearing for nearly a century to grace the mouth of the wearer, and in performing their functions fully give tone to the general system, in as much as the food is masticated properly, not overtaxing the stomach, but allowing it to perform its work with ease.

Cells reproduce cells after their own kind; that is, after the type of the parent cell. A muscle cell will reproduce a muscle cell, yet some claim that a muscle cell under different surroundings will produce other tissue or the pus corpuscle, but this is contrary to the good orthodox doctrine that "every thing shall bear seed after its kind." No one will deny however, that different surroundings will not make a difference in the development of the cell, the same as the difference between a well or ill fed animal or a human being in sickness or in health.

The reproduction of cells is accomplished in several different ways. "First, (Beale L. S. and M. and M. 45.) in many masses of germinal matter, a smaller spherical portion, often appearing a mere point is observed, in some cases this divides before the division of the parent mass takes place. This however, is not necessary to the process, for division takes place in cases in which no such bodies are seen. These are to be regarded as new centers, composed of living matter. Within these a second series is sometimes produced. The first have been called nuclei, and those within them, nucleoli." Some regard these bodies as the only agents of reproduction, this however is not true. This living matter like many other organisms reproduces its kind, not only by germinating and producing seeds or germs; but by throwing out what might be termed runners, and when these runners are sufficiently strong to sustain their own life without the aid of the parent, the connection between them and the parent germinal matter is severed; then they become separate living cells capable of reproducing their kind, and fulfilling all the functions of the parent cell.

The reproduction of cells is far more rapid in the young and fast growing. The rapidity of reproduction diminishes as age advances. The rapidity of reproduction depends also upon the surroundings, such as variations of temperature from one extreme to another; the material of which the pabulum is formed; the presence of malarial poisons or contagious disease germs. In inflammation and fever (according to Beale) there seems to be an excessive development and reproduction of germinal matter.

It is claimed by some that the excessive development of the germinal matter is the cause of diseases, yet it seems far more probable, that it is owing to the state of the pabulum, the presence of poisons or the presence of germs of contagion. In illustration of the first, (excessive development in inflammation and fevers) if when we are in profuse perspiration, we become suddenly chilled, it may throw the system into violent inflammation or fever, it is said that it is caused by closing the pores of the skin, but it seems far more probable that the germinal matter in the cells which line the perspiratory glands which separates or extracts the poisonous gasses or fluids from the blood have ceased to act, and perhaps a large portion of the germinal matter has died altogether from the extreme change of temperature; this takes away the means of eliminating the poisons. The retention of these poisons in the system seems to destroy the action or function of the germinal matter which is to build up tissue; hence we observe a rapid wasting away of the body. This retention also seems to have the effect, (from the previous reference I made to Beale) of increasing the reproductive powers of the germinal matter of the tissues of the body; but whether it is the reproduction of the germinal matter of the tissues of the body or the germinal matter of organisms foreign to the tissues in which they are found, may be questioned. It is well known that there are myriads of germs floating in the pabulum which have penetrated into every portion of the human system, only waiting for the proper condition of the surroundings to bring the germ into living germinal matter, and, from what little observation I have had, it seems that all they want is that a portion of the tissues or body be brought to the putrefactive stage, when they quickly assume the living form, thereby furthering the process of decomposition, by which, the process of elimination from the system is hastened.

In inflammation of the periosteum dentium we have a similar occurrence. First, there is an injury of the periosteal tissue; it may be mechanical, or it may arise from the action of poisonous gasses or fluids which come from putrefying substances in the pulp chamber; the injury causing death of a portion of the germinal matter at the apex of the root. As soon as death occurs, it immediately assumes the putrefactive stage and the development of the germs of the pus corpuscle, which seems to be present in nearly all animal tissue, follows.

Within certain bounds the reproduction of germinal matter may go, either above or below the normal standard without material injury to the system, but carry it to either extreme the result is disease.

Different medicines have different effects on the reproduction of germinal matter, those classed under the head of tonics have the property of hardening tissue and making it less permeable to the fluids. They also have an antiseptic property as has also carbolic acid, wood creosote, chloride of zinc, tanin, alcohol, etc., hence the benefit we derive from their use in treating ulcerated teeth and other diseases of the associate tissues.

To increase the reproduction of germinal matter, when below the normal standard (Beale says) "potash, soda, lithia and their carbonates, as well as the salts of many vegetable acids which become converted into carbonates in the system act beneficially in this way as well as by producing favorable changes of other kinds.

It may seem that much I have said has nothing to do with dental physiology, yet upon the physiological condition of the cell, life either ascends or descends in the scale of health; and until we can understand or know the normal condition of the cell, and the action of different remedies upon it to restore it to its normal condition we have not obtained that standard of medical education which should be the desire of every student of the profession.

